# Therapeutic Value of Low-Dose Acetylsalicylic Acid for the Prevention of Preeclampsia in High-Risk Bulgarian Women

**DOI:** 10.7759/cureus.66298

**Published:** 2024-08-06

**Authors:** Boris Stoilov, Ekaterina Uchikova, Zlatko Kirovakov, Polina Zaharieva-Dinkova

**Affiliations:** 1 Obstetrics and Gynaecology, Medical University Plovdiv, Plovdiv, BGR; 2 Midwifery Care, Faculty of Health Care, Medical University Pleven, Pleven, BGR

**Keywords:** acetylsalicylic acid, hypertension, outcome, preterm, preeclampsia

## Abstract

Introduction

Preeclampsia (PE) is a syndrome that affects pregnant women after 20 weeks of gestation and involves numerous organ systems. Screening for PE is essential to prevent complications and guide management. Some existing guidelines for screening have limitations in terms of detection rates and false positives. The aim of this study is to assess the therapeutic value of low-dose acetylsalicylic acid (ASA) for the prevention of PE in high-risk Bulgarian women.

Methodology

A prospective cohort research was carried out, encompassing women who were recruited from several routine consultations, such as booking, scanning, and regular prenatal visits. We utilized the purposive sampling technique to carefully choose potential participants. The study was conducted by a maternal-fetal medicine center located in Plovdiv, Bulgaria. The data-gathering period spanned from January 2018 to November 2020. At the appointment, the following procedures were conducted: 1) recording history; 2) assessing height, weight, and blood pressure; 3) collecting blood specimens for biochemical markers; and 4) ultrasound examination.

Results

A total sample size of 1,383 individuals was categorized into two distinct groups: high-risk patients (n = 506) and low-risk patients (n = 877). The mean uterine artery pulsatility index (UtA-PI) and mean arterial pressure (MAP) ratios were all greater in high-risk group women (p < 0.05). The data revealed that a significant number of high-risk women failed to adhere to the prescribed dosage or regular use of ASA as recommended by their doctor. There were only 384 (75.9%) high-risk women who took low-dose ASA regularly.

Conclusion

The findings emphasize the importance of personalized prenatal care and early risk assessment to improve maternal and fetal outcomes. Therefore, it is crucial to educate pregnant women, considering the benefits and risks of low-dose ASA when appropriately indicated.

## Introduction

Preeclampsia (PE) is a syndrome that affects pregnant women after 20 weeks of gestation and involves numerous organ systems. It is classified as either superimposed maternal hypertension or de novo hypertension and proteinuria [1]. In some underdeveloped countries, the prevalence reaches between 10% and 18%, accounting for approximately 2%-3% of pregnancies with PE [2]. Early-onset PE commonly causes foetal growth restriction (FGR) and adverse outcomes for both mothers and babies. Late-onset PE, on the other hand, is associated with better perinatal outcomes, fewer severe symptoms for the mother, and a lower chance of foetal problems [3].

The Foetal Medicine Foundation (FMF) algorithm, which considers maternal factors such as mean arterial pressure (MAP), uterine artery pulsatility index (UtA-PI), and placental growth factor (PlGF), consistently outperforms the National Institute for Health and Clinical Excellence (NICE) and American College of Obstetricians and Gynaecologists (ACOG) recommended methods for PE screening between the 11^th^ and 13^th^ weeks of gestation [4]. The NICE guideline has a 10% false-positive rate and detection rates of 41% for preterm and 34% for term PE [3], whereas the FMF algorithm has high detection rates of 90% and 75% for early and late PE prediction, respectively with a 10% false-positive rate. Furthermore, the plan recommends assessing the risk in the second and third trimesters of pregnancy [5].

The ocular artery is an accessible channel for Doppler examination, providing information about the less accessible cerebral circulation [6]. It resembles the brain vasculature in both architecture and function [7, 8]. Ocular issues may occur in 30% to 100% of women with PE [9-11]. The ocular Doppler is a reliable and unbiased approach for evaluating how serious PE is. It is an accessible vessel with the potential for PE screening. Recent research suggests that early detection of peripheral artery disease (PAD) by ocular artery screening has great promise for rapid intervention and therapy [12]. Despite these variables, the prevalence of PE has remained rather stable during the last few decades [13]. Numerous highly diverse studies have evaluated the potential benefit of taking acetylsalicylic acid (ASA) during pregnancy to reduce the risk of PE; these studies differed greatly in terms of the demographic risk profile including the ASA dosage, the gestational age at which prophylactic treatment was initiated, and the disease definition [14-18]. When administered to high-risk women at doses larger than 100 mg and begun before 16 weeks of gestation, ASA is particularly effective in preventing preterm PE, lowering its incidence by more than 60%. As a result, the first trimester of pregnancy is appropriate for identifying high-risk women, preferably using prediction algorithms [19].

Taking ASA at 150 mg daily beginning before 16 weeks of gestation and administered at night to a high-risk population identified by a combined first trimester screening test has been shown to lower the incidence of preterm PE by 62%. These data are based on the Aspirin for Evidence-Based Preeclampsia Prevention (ASPRE) study. A secondary assessment of data from the ASPRE study revealed that when compared to placebo, the length of stay in the newborn intensive care unit was reduced by 68%. This reduction was primarily attributable to a decrease in preeclamptic deliveries before 32 weeks of gestation [19]. The Bulgarian Society of Obstetrics and Gynaecology recommends that all women with a risk of PE greater than 1:150, based on the FMF algorithm, begin taking ASA 150 mg every night before 16 weeks until 34 weeks for prevention [20]. Despite the guidelines, we continue to see inadequate levels of screening and prevention for PE, with a 5.3% incidence [21]. It's crucial to understand modern prenatal screening tools and how to prevent various issues. Unfortunately, the most advanced methods of prenatal care in Bulgaria are not supplied by the government, and patients must take care of themselves. Providing local facts could help to awaken society. Preeclampsia screening and care aim to reduce perinatal problems for expecting mothers and newborns by initiating preventative actions for high-risk groups or determining the optimal delivery time [22]. The aim of this study is to assess the therapeutic value of low-dose ASA for the prevention of PE in high-risk pregnancies among Bulgarian women.

## Materials and methods

Study design and participants

A prospective cohort study was carried out encompassing women who were recruited from several routine consultations such as booking, scanning, and regular prenatal visits. We utilised the purposive sampling technique to carefully choose potential participants. We employed a dual set of conditions for inclusion: a solitary pregnant individual who was a minimum of 18 years of age and devoid of significant mental and physical ailments with a foetus measuring between 45 and 84 mm and a feasible pregnancy duration ranging from 11 weeks + 0 days to 13 weeks + six days. The criteria for exclusion were as follows: the study did not include stillbirths and focused on many factors such as minors, multiple pregnancies, coagulation disorders, foetal abnormalities, gastritis and ulcers, abortion, sensitivity to ASA, and termination of pregnancy.

The study was conducted by a maternal-foetal medicine centre located in Plovdiv, Bulgaria. The data-gathering period spanned from January 2018 to November 2020. In total 1,730 women volunteered to participate in the survey; however, 347 of them subsequently withdrew due to incomplete medical records. Prior to implementing any interventions, the obstetrician conducting the study assessed each woman and confirmed the health and safety of both the mother and the foetus. All non-invasive tests and ultrasounds conducted within the cohort adhered to a standardised protocol and were conducted in a consistent location and administered by the same operator.

**Figure 1:**
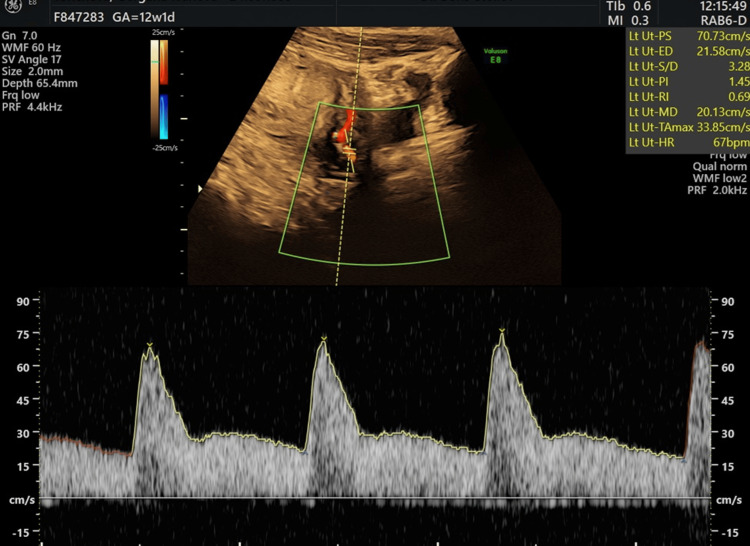
Doppler study: pulsatility index (PI) uterine artery (original image from the study)

At the 11-13 week appointment, the following procedures were conducted: 1) recording history; 2) assessing height, weight, and blood pressure; 3) collecting a blood specimen for biochemical markers at the first trimester scan: free beta-human chorionic gonadotropin (hCG), pregnancy-associated plasma protein-A (PAPP-A), and PlGF (used for PE screening); and 4) ultrasound examination-Doppler study for measurement of pulsatility index of both uterine arteries (Figure 1).

The total sample size of 1383 individuals was categorised into two distinct groups: high-risk patients (n = 506) and low-risk patients (n = 877). Patients were classified into two groups: high-risk (with a PE risk greater than 1:150) or low-risk (with a PE risk less than 1:151). Following the delivery, data were gathered to assess the result and various issues such as FGR, gestational hypertension (GH), diabetes, or any other disorder that began to show. The study's objectives have been effectively met by considering the inclusion and exclusion criteria.

Low-dose ASA intake

Every patient who was defined as “high risk” for developing preterm PE based on the FMF algorithm was advised to start ASA 150 mg at bedtime from 12 weeks till 36 weeks of gestation. The ASA should be started before 16 weeks of gestation; otherwise, there is a lack of effect and an increased risk of abruption of the placenta.

Outcome measures

The evaluation of PE outcomes was conducted according to the International Society for the Study of Hypertension in Pregnancy (ISSHP) recommendations. These criteria indicate that the systolic blood pressure was higher than 140 mm Hg or the diastolic blood pressure was higher than 90 mm Hg on two separate occasions with a time interval of at least four hours occurring after 20 weeks of pregnancy in women who previously had normal blood pressure. Additionally, at least one of the following conditions must be present: (1) Renal insufficiency characterised by a serum creatinine level greater than 97 µmol/L without any other kidney disease; (2) hepatic dysfunction indicated by elevated blood transaminase levels; (3) proteinuria defined as a 24-hour urinary protein excretion of 300 mg or higher, a protein to creatinine ratio over 30 mg/mmoL, or a positive result (2+) on dipstick testing.

The participants were categorised into two groups based on the pregnancy outcome: Group 1 consisted of patients who developed PE, and Group 2 consisted of patients who did not develop PE. The severity of pulmonary embolism in group one was not assessed due to the presence of only one patient in this group who exhibited characteristics of severe PE, including HELLP (haemolysis, elevated liver enzymes, low platelet count) syndrome.

Ethical considerations

The study received approval from the ethics committee of the Medical University-Plovdiv (institutional review board (IRB) number 723) and was carried out in compliance with the Declaration of Helsinki and the principles of Good Clinical Practice. All cases were collected with written informed consent. Prior to the analyses, all records underwent anonymisation and de-identification.

Data analysis

Version 4.2.2 of the R software (2022-10-31 ucr, The R Core Team, R Foundation for Statistical Computing, Vienna, Austria) processed the data, and an α level of 0.05 was used to determine significance. Several statistical analyses are used, such as graphical analysis, one-way ANOVA (analysis of variance) with either the least significant difference (LSD) or for comparison of multiple intergroup Dunnett's T3, chi-squared test (X2 analysis), and descriptive analysis. The selected statistical methods are well-suited for addressing this study's research questions and objectives. They allow for an in-depth analysis of the relationship between low-dose ASA intake and pregnancy-related conditions and the impact of factors like timing of intake and compliance. Using a combination of statistical methods provides a comprehensive view of the data and can help draw meaningful conclusions and insights from the research.

## Results

There were a total of 1,383 pregnant women who participated in the study. The mean age of the participants was 30.1 ± 5.1 years, with a range of 18-47 years. The average gestational age of the participants was 12.4 ± 0.6 weeks. While only 160 (11.6%) women smoked, 53 (3.8%) of them were diagnosed with gestational diabetes mellitus (GDM). The majority of the women had spontaneous conception. The statistically significant differences between the high-risk and low-risk groups were observed in relation to a number of variables that are shown in Tables 1-2 (p <0.05). Our results find that the most significant sociodemographic variables were BMI, conception method, chronic diseases, and interval between pregnancies. With the increase in the caesarean section rate in Bulgaria (recently around 525 (59.9%)), it was not surprising that most of the women with high risk for PE had operative birth.

**Table 1:** Sociodemographic variables *BMI: body mass index; gw: gestational weeks; GA: gestational age; *represents a significant p-value

Variables	Low risk		High risk		F	p-value
	n	mean±StD	n	mean±StD		
Age	877	29.8±4.9	506	30.3±5.5	2.73	0.099
BMI 11-14 gw (kg/m^2^)	877	23.3±4.3	506	25.7±5.7	75.65	0.000*
Interval between births	877	2.8±3.8	506	1.8±3.9	20.41	0.000*
Outcome GA in this pregnancy	877	38.4±2.3	506	38.1±2.5	8.58	0.003*

**Table 2:** Risk factor variables GDM: gestational diabetes mellitus; C-section: caesarean section; PE: preeclampsia; PIH: pregnancy-induced hypertension; FGR: foetal growth restriction; * represents a significant p-value

Variables		n	%	n	%	Chi-square	p-value
Anti-hypertensive medication	No	833	95	442	87.4	25.96	0.000*
	Yes	44	5	64	12.6		
Smoking currently	No	774	88.3	449	88.7	0.722	0.788
	Yes	103	11.7	57	11.3		
Conception	Spontaneous	850	96.9	477	94.3	5.811	0.016*
	In vitro fertilization	27	3.1	29	5.7		
GDM	No	845	96.4	485	95.8	0.219	0.640
	Yes	32	3.6	21	4.2		
Chronic hypertension	No	871	99.3	489	96.6	14.05	0.000*
	Yes	6	0.7	17	3.4		
Delivery	Birth	352	40.1	153	30.2	13.57	0.000*
	C-section	525	59.9	353	69.8		
PE/PIH	Without PE/PIH	828	94.4	433	85.6	33.52	0.000*
	PE	10	1.1	24	4.7		
	PIH	39	4.4	49	9.7		
FGR	No	837	95.4	452	89.3	18.95	0.000*

As expected, the mean UtA-PI and MAP ratio were all greater in the high-risk group women (p <0.05) (Table 3). The most relevant biochemical marker was found to be placental growth factor (PlGF) in our results. The high-risk women had lower PlGF compared to the low-risk patients. It is seen with PAPP-A as well but not so significantly as with the PlGF. 

**Table 3:** Preeclampsia biomarkers PlGF: placental growth factor; MoM: multiple of the media; PAPP-A: pregnancy-associated plasma protein-A; MAP: mean arterial blood pressure; mean UtA-PI: mean pulsatility index of uterine arteries; * represents a significant p-value

Variables	Patient groups	n	Mean	Std. deviation	F	p-value
PlGF MoM	Low risk	877	1.0	0.5	323.67	0.000*
	High risk	506	0.6	0.3		
	Total	1383	0.9	0.5		
PAPP A MoM	Low risk	877	1.1	0.6	32.62	0.000*
	High risk	506	0.9	0.5		
	Total	1383	1.1	0.6		
MAP	Low risk	877	88.1	7.8	165.46	0.000*
	High risk	506	93.8	8.1		
	Total	1383	90.1	8.4		
Mean UtA-PI	Low risk	877	1.8	0.5	141.11	0.000*
	High risk	506	2.1	0.5		
	Total	1383	1.9	0.5		

There were only 384 (75.9%) high-risk women who took ASA regularly. Although their dosage varied, the majority of the women took 150 milligrams of ASA per night. They began the intake at approximately 12.4 ± 1.0 weeks of gestation and continued it until approximately 34.6 ± 3.9 weeks of gestation. Some of the high-risk women (38, 7.5%) were inconsistent with the intake of the medication. The explanation was that they were not reassured that ASA was going to help or because of the time of the prescription that was normotensive (Table 4).

**Table 4:** Low-dose acetylsalicylic acid (ASA) intake in women at high risk

Variables		High risk	
		n	%
ASA intake	Do not take	84	16.6
	Irregular intake	38	7.5
	Regular intake	384	75.9
Dosage (mg)	Do not take	84	16.6
	Less than 150 mg	16	3.2
	150 mg	406	80.2

**Figure 2:**
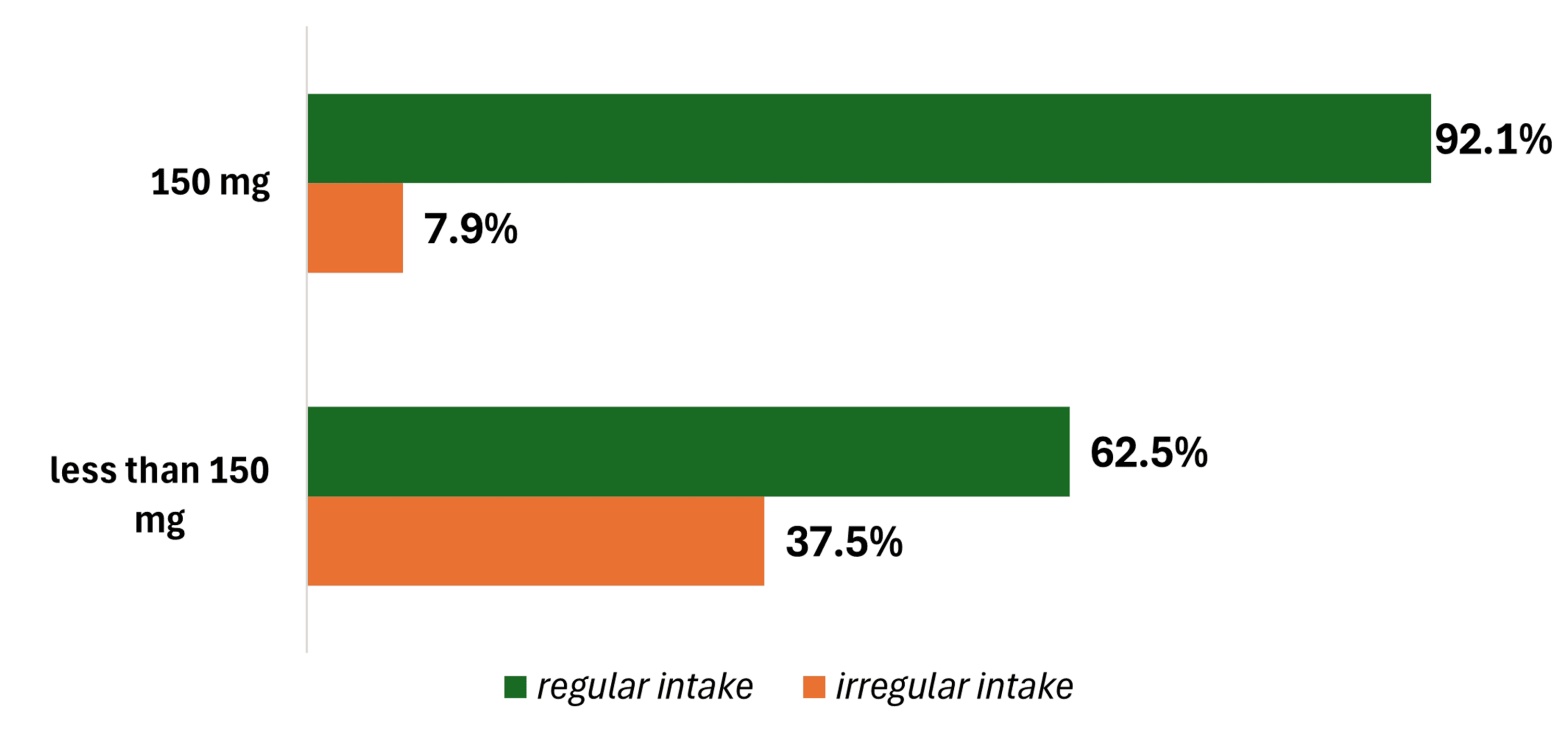
Regular or irregular intake of recommended low-dose acetylsalicylic acid (ASA)

Although 506 high-risk patients used low doses of ASA, only 22 (5.4%) developed PE and 49 (9.7%) had pregnancy-induced hypertension (PIH) after taking the medication. Of those taking ASA 150 mg regularly, only 21 (5.6%) developed PE. It is expected that high-risk patients will develop that complication. Only 84 (16.6%) of high-risk patients declined to take the medication. Although the remaining 16 participants (3.2%) consumed less than 150 mg, only 10 (62.5%) maintained a consistent intake of that dosage. It is noteworthy that 32 (7.9%) of high-risk women who took the adequate dosage of 150 mg did not consistently adhere to the medication regimen (Figure 2). Patients of advanced age consumed ASA irregularly or not at all (p = 0.050). Furthermore, women who had in vitro fertilisation did not comply with the recommended dosage of ASA (p = 0.027).

## Discussion

The main focus in the battle against PE is its successful early prediction [5], which is a leading scientific motive to re-evaluate the adopted prediction models, look for new indicators for the PE risk [12], and its related additional difficulties such as premature birth, FGR, and others. The association between PE and GH in pregnant women, low-dose ASA intake, and risk assessment is thoroughly analysed in this study. According to the survey, regular low-dose ASA consumption is linked to a decreased risk of PE and GH in comparison to sporadic or non-aspirant consumption [19]. This suggests a potential protective effect of regular ASA use in expectant women. Relative to the control group, pregnant women who received the intervention, screening, and subsequent prevention for high-risk patients for different gestational weeks had significantly lower incidences of developing PE. The reduction in PE incidence was more pronounced in women who started preventive measures earlier in pregnancy. The study found that the earlier the PE developed in pregnancy, the higher the predictive power of risk assessment. Early-onset PE is often associated with more severe forms of the condition, making it more detectable through risk assessment tools. Most women in the study took 150 mg of low-dose ASA. Within the group of individuals at high risk, compliance with regular low-dose ASA intake was comparatively high. This implies that many expectant mothers are prepared to follow preventive guidelines.

The combination of maternal factors and these three biomarkers can reliably predict approximately 85% of PE cases before 34 weeks and 75% of cases before 37 weeks, emphasising the vital importance of assessing PIGF, UtA-PI, and MAP as early indicators of PE. Screening tests that consider the pulsatility index of the uterine artery, arterial blood pressure, and levels of placental proteins, like PAPP-A and PlGF, offer promising methods for PE risk assessment [23]. Our data reviews very similar results and finds MAP, PlGF, UtA-PI, BMI in the first trimester, and conceptional method (spontaneous and in vitro fertilisation (IVF)) as the most reliable markers for the prediction of preterm PE. A significant number of pregnant women at a high risk of early-onset PE can be identified as early as 11-13 weeks of gestation using a novel mathematical model known as Bayes' theorem. This model incorporates maternal factors and histories such as medical and obstetric, uterine artery PI, PIGF serum levels, MAP, and maternal PAPP-A to enhance early detection [24].

Similarly, by utilising the Bayes' theorem and considering maternal factors along with MAP, UtA-PI, and serum PlGF levels, as mentioned, it became possible to predict the occurrence of PE at different stages of pregnancy [5, 25, 26]. With a 10% false positive rate, this approach allowed for the prediction of PE with a level of 75% (with a 95% confidence interval ranging from 62% to 85%) before 37 weeks of gestation, 43% (with a 95% confidence interval ranging from 35% to 50%) after 37 weeks, and a remarkable 100% (with a 95% confidence interval ranging from 80% to 100%) before 32 weeks. Established during the 11-13 weeks of gestation, these findings indicate an efficient method for early PE screening in the first trimester, as concluded by the authors [5, 27].

Compliance seems to be the key to ASA's protective effect in the prevention of preterm PE [28]. The compliance with ASA in our study was 80.6%. Although extended explanations to the patients regarding the importance of PE prophylaxis based on the results of the ASPRE trial, there was hesitation and even hostility among patients. Even though the results of our study showed a reduction in PE incidence, inadequate information, failure to identify risk factors, and attitude regarding the negative effects of taking medicine were found to be associated with other domains, including the environmental context, and to be consistent with the Necessity-Concerns Framework [29].

Nevertheless, we need to focus our efforts on promoting the screening and prevention of PE in Bulgaria by not only explaining to the patients but, most importantly, to the healthcare providers. Some of our patients disclosed that the ASA was withdrawn by their maternity healthcare provider. Obviously, the compliance of the patients is good, but we can do much more with the support of every physician. There are guidelines for PE in Bulgaria but with certain leak points. Screening at the first trimester is partially provided by the government, and PE is not included. The lack of regulations for healthcare providers is a major problem. There is no clear rule for license and audit. Nevertheless, of the local limitations, the screening and prevention of PE are well accepted by the majority of the patients [21, 22]. 

These findings support the long-standing trend that early PE is better suited for the predictive models adopted to assess PE risk. In the study, we examined the effectiveness of low-dose ASA given at night from 12 weeks to 36 weeks for high-risk group women who underwent screening for PE between 11+0 and 13+6 weeks. We measured the effect by using a group of patients who did not have screening for PE, and some were taking ASA without clear indications, dose, or period. The study outcome proved low-dose ASA effectiveness in preventing PE for high-risk groups starting from the end of the first trimester until 36 gestational weeks. The results strongly support low-dose ASA use as a preventive measure for PE, especially in high-risk pregnant women. Early initiation of preventive strategies appears more effective in reducing PE incidence [17, 18, 28].

Moreover, the findings suggest that healthcare providers should consider risk assessment as a routine part of prenatal care, allowing for early identification of high-risk pregnancies and enabling targeted interventions and better outcomes for mothers and their infants [1-3, 5, 7, 12]. The findings also stress the importance of personalised care and adapting preventive measures during pregnancy to individual risk profiles, improving maternal and foetal outcomes [19]. Likewise, by reducing the incidence of PE through screening and prevention, healthcare resources can be allocated more efficiently, potentially reducing the burden on healthcare systems. Furthermore, the study recommends that pregnant women receive education and guidance on the benefits and risks of low-dose ASA intake. Prior to pregnancy, during the preconception period, it is imperative that women at high risk are provided with knowledge regarding the benefits of consuming low-dose ASA to avoid PE. This information should be obtained mostly from web-based educational platforms [30]. Regular monitoring of their health and risk factors is essential for early detection and timely intervention [20, 21, 28].

Limitations

One of the most significant limitations of this study is the limited sample size that was analysed, which has an impact on the sensitivity and specificity of the screening for PE in pregnant women. It is also essential to point out that this study was conducted at a single centre, which eliminates the possibility of making a comparison to a more extensive study.

## Conclusions

Screening for PE is essential to prevent complications and guide management. Some existing guidelines for screening have limitations in terms of detection rates and false positives. The data revealed that a significant number of women fail to adhere to the prescribed dosage or regular use of ASA as recommended by their doctor. Early screening and preventive measures significantly reduced the occurrence of PE. The findings emphasise the importance of personalised prenatal care and early risk assessment to improve maternal and foetal outcomes. Therefore, it is crucial to educate pregnant women, considering the benefits and risks of low-dose ASA when appropriately indicated. This study highlights the potential benefits of low-dose ASA and early risk assessment in preventing PE and its associated complications during pregnancy.
